# Effect of Device Size on Outcomes Following Left Atrial Appendage Closure in Borderline-Sized Anatomy

**DOI:** 10.1016/j.jacasi.2025.11.015

**Published:** 2026-01-21

**Authors:** Kensuke Kuwabara, Shunsuke Kubo, Yoshifumi Nakajima, Takashi Matsumoto, Masato Fukunaga, Yusuke Kondo, Hiroki Sugane, Kenji Okubo, Koji Nakagawa, Daisuke Nagatomo, Daisuke Hachinohe, Shigeki Kusa, Tomoyuki Umemoto, Mamoru Nanasato, Takeshi Arita, Hiro Yamasaki, Tomotaka Yoshiyama, Nobuaki Tanaka, Masaharu Masuda, Tomohiro Sakamoto, Masaki Nakashima, Yohei Ohno, Shigeru Saito, Hidehiko Hara

**Affiliations:** a Department of Cardiology, Kishiwada Tokushukai Hospital, Kishiwada, Japan; b Department of Cardiology, Kurashiki Central Hospital, Kurashiki, Japan; c Division of Cardiology, Iwate Medical University, Morioka, Japan; d Department of Cardiology and Catheterization Laboratories, Shonan Kamakura General Hospital, Kamakura, Japan; e Department of Cardiology, Kokura Memorial Hospital, Kitakyushu, Japan; f Department of Cardiovascular Medicine, Chiba University Graduate School of Medicine, Chiba, Japan; g Division of Cardiology, Chikamori Hospital, Kochi, Japan; h Cardiovascular Center, Yokosuka Kyosai Hospital, Yokosuka, Japan; i Department of Cardiovascular Medicine, Okayama University Hospital, Okayama, Japan; j Division of Cardiology, Saiseikai Fukuoka General Hospital, Fukuoka, Japan; k Department of Cardiovascular Medicine, Sapporo Cardio Vascular Clinic, Sapporo, Japan; l Cardiovascular Center, Tsuchiura Kyodo Hospital, Tsuchiura, Japan; m Department of Cardiovascular Medicine, Institute of Science Tokyo, Tokyo, Japan; n Department of Cardiology, Sakakibara Heart Institute, Tokyo, Japan; o Division of Cardiovascular Medicine, Fukuoka Wajiro Hospital, Fukuoka, Japan; p Department of Cardiology, University of Tsukuba, Tsukuba, Japan; q Department of Cardiovascular Medicine, Osaka Metropolitan University Graduate School of Medicine, Osaka, Japan; r Division of Cardiology, Sakurabashi Watanabe Advanced Healthcare Hospital, Osaka, Japan; s Cardiovascular Center, Kansai Rosai Hospital, Amagasaki, Japan; t Cardiovascular Center Division of Cardiology, Saiseikai Kumamoto Hospital, Kumamoto, Japan; u Division of Cardiology, Sendai Kosei Hospital, Sendai, Japan; v Department of Cardiology, Tokai University School of Medicine, Isehara, Japan; w Division of Cardiovascular Medicine, Toho University Ohashi Medical Center, Tokyo, Japan

**Keywords:** atrial fibrillation, ischemic stroke, left atrial appendage closure, peridevice leak

## Abstract

**Background:**

Selecting the optimal WATCHMAN FLX (a second-generation left atrial appendage [LAA] closure device) size for a borderline-sized LAA, for which 2 device sizes are acceptable, remains challenging.

**Objectives:**

The aim of this study was to evaluate how device size affects procedural and clinical outcomes in borderline-sized LAAs undergoing LAA closure.

**Methods:**

From the Japanese multicenter TERMINATOR (Transcatheter Modification of Left Atrial Appendage by Obliteration with Device in Patients With NVAF) registry, 1,190 patients who underwent LAA closure with the second-generation device between May 2021 and September 2023 were reviewed. Of these, 757 with borderline-sized LAAs were included and categorized into large (n = 390) and small (n = 367) device groups. The median follow-up duration was 274 days (Q1-Q3: 100-368 days). Outcomes included peridevice leak (PDL), all-cause mortality, ischemic cerebrovascular accident or systemic embolism, major bleeding, and device-related thrombus.

**Results:**

PDL occurred less frequently in the large device group compared with the small device group (1.54% [6 of 390] vs 4.90% [18/367]; *P* = 0.015). Ischemic cerebrovascular accident or systemic embolism was lower in the large device group (8.4% [95% CI: 1.8%-34.1%] vs 10.5% [95% CI: 3.6%-28.4%]; *P* = 0.036). No significant difference was observed in all-cause mortality (15.2% [95% CI: 6.0%-35.7%] vs 12.3% [95% CI: 4.6%-30.6%]; *P* = 0.288), major bleeding (4.5% [95% CI: 2.6%-7.7%] vs 15.7% [95% CI: 4.3%-48.7%]; *P* = 0.570), or device-related thrombus (8.6% [95% CI: 3.2%-22.2%] vs 3.2% [95% CI: 1.6%-6.4%]; *P* = 0.603).

**Conclusions:**

In borderline-sized LAAs, the larger device was associated with fewer PDLs and ischemic events without increased procedural risk.

In patients with nonvalvular atrial fibrillation, the left atrial appendage (LAA) is a primary site of thrombus formation,^1^^,^^2^ frequently leading to thromboembolic events such as ischemic cerebrovascular accident (CVA) or systemic embolism. Percutaneous LAA closure (LAAC) using WATCHMAN devices (a plug-type LAAC device; Boston Scientific) has become an effective stroke prevention strategy for patients in whom long-term anticoagulation therapy is unsuitable.^3^, ^4^, ^5^

Numerous trials have demonstrated the efficacy of the LAAC device in preventing thromboembolic events.^4^, ^5^, ^6^, ^7^ However, several factors, including device-related thrombus (DRT) and peridevice leaks (PDLs), compromise postprocedural outcomes.^8^, ^9^, ^10^, ^11^, ^12^ PDLs have been reported to increase the risk for thromboembolic events.^11^^,^^12^ To mitigate these risks and achieve favorable postprocedural outcomes, appropriate device sizing is essential and is typically guided by the manufacturer’s recommendations.

In borderline-sized LAAs, in which the dimensions of the LAA ostium overlap 2 recommended device size ranges, device size selection can be challenging. A study comparing devices that are larger and smaller than the manufacturer’s recommendations reported that larger devices reduce PDLs without increasing complications.^13^ However, no studies have compared large and small devices within the manufacturer-recommended range for borderline-sized LAA.

The aim of this study was to evaluate the effect of device size selection on clinical outcomes in borderline-sized LAAs. Using real-world registry data, we aimed to clarify the distribution and characteristics of borderline-sized cases, current practices of device size selection in clinical settings, periprocedural complications, and postprocedural outcomes. This study may provide crucial insights into optimizing treatment strategies and improving patient outcomes in borderline-sized cases.

## Methods

### Study design

The TERMINATOR (Transcatheter Modification of Left Atrial Appendage by Obliteration with Device in Patients With NVAF) registry is an ongoing multicenter prospective observational cohort study conducted across 23 centers in Japan. This registry includes consecutive LAAC cases performed with the WATCHMAN 2.5 (a first-generation LAAC device), WATCHMAN FLX (a second-generation LAAC device), and WATCHMAN FLX Pro (a third-generation LAAC device). A detailed description of the TERMINATOR registry has been published.^14^ LAAC serves as an alternative to long-term anticoagulation therapy for patients meeting 1 or more of the following criteria for high bleeding risk: a HAS-BLED score of ≥3, a history of multiple falls requiring medical intervention, a history of diffuse cerebral amyloid angiopathy, need for long-term dual antiplatelet therapy, or a history of major bleeding classified as type ≥3 by the Bleeding Academic Research Consortium.^15^

### Study population

**Figure 1:**
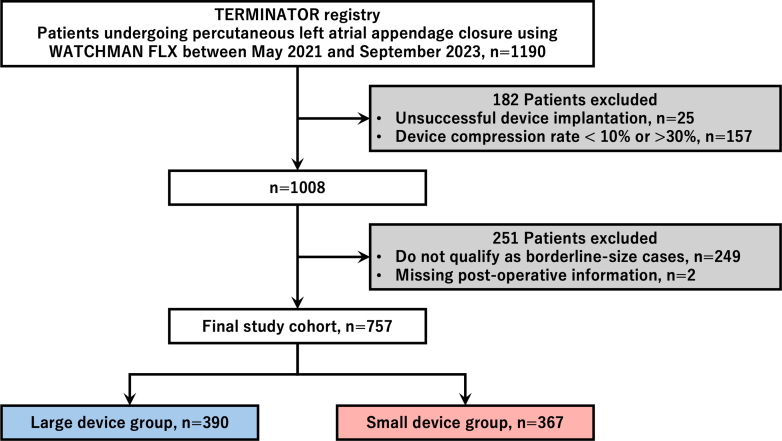
Study Flowchart TERMINATOR = Transcatheter Modification of Left Atrial Appendage by Obliteration with Device in Patients With NVAF.

For this substudy, patients undergoing percutaneous LAAC with the second-generation LAAC device between May 2021 and September 2023 in the TERMINATOR registry were initially screened. Cases of unsuccessful device implantation, postimplantation compression rates outside the manufacturer-recommended range (<10% or >30%), and missing postoperative follow-up data were excluded. Patients with maximal LAA ostial diameters of 16.8 to 18, 18.9 to 21.6, 21.7 to 24.3, and 24.5 to 27.9 mm were categorized as borderline-sized cases, for which 2 device sizes were applicable per manufacturer recommendations. After extracting only borderline-sized cases, the final study cohort consisted of 757 patients (Figure 1).

### Measurement definitions

The maximal and minimal diameters of the LAA ostium were measured using intraprocedural transesophageal echocardiography (TEE) at 0°, 45°, 90°, and 135°, with the ostium defined from the left circumflex coronary artery to a superior point 1 to 2 cm within the pulmonary vein ridge.^16^ The maximal diameter was defined as the largest value across all views, whereas the minimal diameter was the smallest. Then, the deepest measurable LAA depth was obtained from this ostial plane to the distal tip of the appendage.

The appendage eccentricity index was calculated as follows: 1 − (LAA ostial minimal diameter/LAA ostial maximal diameter).^17^ Device compression rate was assessed by measuring the maximal and minimal device diameters on intraprocedural TEE immediately after device release and calculated as follows: (nominal device diameter − measured device diameter)/nominal device diameter × 100. The minimal compression rate was calculated using the maximal measured device diameter, whereas maximal compression rate was calculated using the minimal measured device diameter. No formal inter- or intraobserver reproducibility analyses were conducted for these measurements; however, measurements were obtained by experienced operators at each site.

### Device size selection

In patients with borderline-sized LAAs, the choice between a larger or smaller manufacturer-recommended device was left to the discretion of the implanting operator. The decision was based on a comprehensive assessment of LAA morphology, including ostial size, depth, distal LAA capacity for device accommodation, extent of pectinate muscle trabeculation, and intraprocedural assessment by the implanting operator. No predefined implantation strategy was mandated by the registry.

### Outcomes

The study population was divided into 2 groups according to the size of the implanted device. Patients who were implanted with the larger of the 2 manufacturer-recommended device sizes were placed in the large device group, whereas those implanted with the smaller sizes were categorized into the small device group. In addition, 7.53% of patients (57 of 757) received devices that were either larger or smaller than the manufacturer-recommended sizes; these cases were classified accordingly into the large or small device group.

To evaluate the success of the procedure, technical and procedural success was assessed. Technical success was defined as the successful device implantation with complete exclusion of the LAA and absence of device-related complications and residual leak >5 mm on color Doppler TEE. Procedural success was defined as technical success without any procedure-related complications.^18^ The primary outcomes of interest included all-cause mortality, ischemic CVA or systemic embolism, major bleeding events, DRT, and periprocedural complications.

No independent core laboratory was used for the adjudication of imaging or outcome events. Measurements and event classification were performed at each site according to the prespecified registry definitions.

### Data collection

All centers were mandated to register every case. Data were collected prospectively using standardized case report forms, which included detailed patient demographics, procedural variables, and follow-up outcomes. To ensure consistency across participating centers, these forms capture comprehensive data elements relevant to LAAC device implantation. Data entry was centralized through an electronic data capture system, enabling data validation and minimizing entry errors. Although the TERMINATOR registry follows patients for up to 5 years, this substudy limited the follow-up period to 1.5 years (540 days) to ensure uniformity in data analysis and minimize the effect of cases that had not yet reached longer follow-up time points.

### Statistical analysis

Descriptive analyses summarized the baseline characteristics, procedural details, and clinical outcomes. Depending on the data distribution, continuous variables are expressed as mean ± SD or medians (Q1-Q3), whereas categorical variables are expressed as frequencies and percentages. Intergroup comparisons for continuous variables were performed using Student’s *t*-test for parametric variables or the Mann-Whitney *U* test for nonparametric variables. Categorical variables were compared using the chi-square test or the Fisher exact test, as appropriate. The Kaplan-Meier method was used to estimate the cumulative rates of all-cause mortality, ischemic CVA or systemic embolism, major bleeding events, or DRT in the 2 groups stratified by device size. Survival differences between the groups were compared using log-rank tests. A univariate Cox regression analysis was performed to obtain the HRs for ischemic CVA or systemic embolism. The proportional hazards assumption was assessed using Schoenfeld residuals. For variables that violated this assumption (male sex and prior systemic embolization), time-stratified Cox models were applied, with follow-up divided at 180 days (early vs late phases) to account for nonproportional hazards. All statistical tests were 2-sided, and *P* values <0.05 were considered to indicate statistical significance. All statistical analyses were performed using R version 4.4.2 (R Development Core Team).

### Ethical approval

This study was approved by the Institutional Review Boards of all participating institutions and conducted in accordance with the principles outlined in the Declaration of Helsinki. The study protocol was registered in the University Hospital Medical Information Network Clinical Trials Registry (UMIN000044934). Before enrollment, all participants provided informed consent.

## Results

### Study population and baseline characteristics

The study included a total of 757 patients, with a median age of 78.0 years (Q1-Q3: 73.0-82.0 years), and 68.3% (517 of 757) were men. The median CHA_2_DS_2_-VASc and HAS-BLED scores were 5 (Q1-Q3: 4-6) and 3 (Q1-Q3: 3-4), respectively.

Intraprocedural crossover between device groups occurred in 36 cases: a crossover from the initially assigned small device group to the large device group was required in 28 of 387 patients (7.2%), whereas a crossover from the initially assigned large device group to the small device group was necessary in 8 of 370 patients (2.2%). The initial assignment to the small device group was significantly more likely to require crossover than the initial assignment to the large device group (*P* = 0.001).

The baseline characteristics were generally well balanced between the 2 groups (Table 1). However, the proportion of patients with permanent or persistent atrial fibrillation was significantly higher in the large device group than in the small device group (63.59% [248 of 390] vs 50.14% [184 of 367]; *P* = 0.001). In addition, the incidence of prior systemic embolism was significantly higher in the large device group than in the small device group (11.79% [46 of 390] vs 7.08% [26 of 367]; *P* = 0.037).

**Table 1:** Baseline Patient Characteristics

	Overall (N = 757)	Large Device Group (n = 390)	Small Device Group (n = 367)	*P* Value
Age, y	78.00 (73.00-82.00)	78.00 (73.00-82.00)	78.00 (73.00-82.00)	0.789
Male	517 (68.30)	259 (66.41)	258 (70.30)	0.284
BMI, kg/m^2^	23.00 (20.80-25.70)	22.80 (20.70-25.60)	23.50 (20.90-25.75)	0.322
Permanent or persistent AF	432 (57.07)	248 (63.59)	184 (50.14)	0.001
CHADS_2_ score	3 (2-4)	3 (2-4)	3 (2-4)	0.038
CHA_2_DS_2_-VASc score	5 (4-6)	5 (4-6)	5 (4-6)	0.532
HAS-BLED score	3 (3-4)	3 (3-4)	3 (3-4)	0.316
Hypertension	622 (82.17)	317 (81.28)	305 (83.11)	0.575
Diabetes mellitus	264 (34.87)	128 (32.82)	136 (37.06)	0.252
Prior PCI	191 (25.23)	96 (24.62)	95 (25.89)	0.750
Prior CABG	35 (4.62)	22 (5.64)	13 (3.54)	0.230
Prior MI	100 (13.21)	52 (13.33)	48 (13.08)	1.000
Peripheral artery disease	99 (13.08)	55 (14.10)	44 (11.99)	0.451
Carotid stenosis	50 (6.61)	25 (6.41)	25 (6.81)	0.939
NYHA functional class (III or IV)	21 (2.77)	14 (3.59)	7 (1.91)	0.235
Prior ablation	262 (34.61)	133 (34.10)	129 (35.15)	0.821
Prior hemorrhagic stroke	113 (14.93)	53 (13.59)	60 (16.35)	0.336
Prior ischemic stroke	323 (42.67)	161 (41.28)	162 (44.14)	0.471
Prior systemic embolization	72 (9.51)	46 (11.79)	26 (7.08)	0.037
Prior major bleeding	283 (37.38)	147 (37.69)	136 (37.06)	0.916
Any cancers	189 (24.97)	93 (23.85)	96 (26.16)	0.818
Steroid use	29 (3.83)	11 (2.82)	18 (4.90)	0.192
LVEF, %	60.70 (54.38-65.05)	60.00 (53.10-65.00)	62.00 (55.75-66.00)	0.035
Hemoglobin, g/dL	12.30 (10.90-13.72)	12.20 (10.90-13.60)	12.50 (11.00-13.80)	0.325
Platelet count, ×1,000/μL	187.00 (150.00-224.75)	182.00 (150.00-222.00)	194.00 (150.00-227.00)	0.149
Albumin, g/dL	3.80 (3.60-4.10)	3.90 (3.60-4.10)	3.80 (3.50-4.10)	0.509
eGFR, mL/min/1.73 m2	52.04 (32.84-66.64)	51.97 (31.48-65.95)	52.06 (34.01-67.00)	0.333

Values are medians (Q1-Q3) or n (%).

AF = atrial fibrillation; BMI = body mass index; CABG = coronary artery bypass graft; eGFR = estimated glomerular filtration rate; LVEF = left ventricular ejection fraction; MI = myocardial infarction; PCI = percutaneous coronary intervention.

### Device selection and procedural outcomes

The study population was categorized into 4 anatomical zones according to the maximal diameters of the LAA ostium: zone 1 (16.8-18 mm), zone 2 (18.9-21.6 mm), zone 3 (21.7-24.3 mm), and zone 4 (24.5-27.9 mm). Table 2 summarizes the device use and distribution of LAA dimensions between the 2 groups.

**Table 2:** Device Use and Distribution of Left Atrial Appendage Dimensions Across Groups

	Overall	Large Device Group	Small Device Group	*P* Value
Zone 1	N = 34	n = 26	n = 8	
Number of devices used	1 (1-1)	1 (1-1)	1 (1-1)	0.710
Device size				
20 mm	8	0	8	NA
24 mm	22	22	0	NA
27 mm	3	3	0	NA
31 mm	1	1	0	NA
LAA maximum diameter, mm	17.38 ± 0.40	17.45 ± 0.39	17.14 ± 0.38	0.065
LAA minimum diameter, mm	13.96 ± 1.92	14.22 ± 1.56	13.03 ± 2.81	0.319
LAA depth, mm	20.01 ± 4.65	19.93 ± 4.54	20.23 ± 5.24	0.891
Zone 2	N = 187	n = 126	n = 61	
Number of devices used	1 (1-1)	1 (1-1)	1 (1-1)	0.054
Device size				
20 mm	2	0	2	NA
24 mm	59	0	59	NA
27 mm	101	101	0	NA
31 mm	22	22	0	NA
35 mm	3	3	0	NA
LAA maximum diameter, mm	20.35 ± 0.79	20.56 ± 0.74	19.92 ± 0.72	<0.001
LAA minimum diameter, mm	16.32 ± 2.15	16.66 ± 2.08	15.65 ± 2.16	0.003
LAA depth, mm	23.14 ± 5.50	23.28 ± 5.77	22.85 ± 4.92	0.60
Zone 3	N = 276	n = 136	n = 140	
Number of devices used	1 (1-1)	1 (1-1)	1 (1-1)	0.108
Device size				
20 mm	1	0	1	NA
24 mm	4	0	4	NA
27 mm	135	0	135	NA
31 mm	122	122	0	NA
35 mm	14	14	0	NA
LAA maximum diameter, mm	23.02 ± 0.77	23.33 ± 0.70	22.71 ± 0.71	<0.001
LAA minimum diameter, mm	18.47 ± 2.39	19.19 ± 2.04	17.75 ± 2.50	<0.001
LAA depth, mm	23.99 ± 6.10	24.56 ± 5.83	23.45 ± 6.32	0.135
Zone 4	N = 260	n = 102	n = 158	
Number of devices used	1 (1-1)	1 (1-1)	1 (1-1)	0.822
Device size				
24 mm	1	0	1	NA
27 mm	6	0	6	NA
31 mm	151	0	151	NA
35 mm	102	102	0	NA
LAA maximum diameter, mm	26.06 ± 1.00	26.66 ± 0.94	25.67 ± 0.83	<0.001
LAA minimum diameter, mm	20.77 ± 2.58	21.39 ± 2.64	20.38 ± 2.48	0.003
LAA depth, mm	26.33 ± 5.71	26.95 ± 5.47	25.95 ± 5.84	0.172
All zones	N = 757	n = 390	n = 367	
LAA eccentricity index	0.19 (0.13–0.26)	0.18 (0.12–0.25)	0.20 (0.14–0.27)	0.002
Maximum compression rate, %	20.45 ± 4.38	21.22 ± 4.34	19.63 ± 4.28	<0.001
Minimum compression rate, %	15.20 ± 3.84	15.86 ± 4.02	14.51 ± 3.50	<0.001

Values are median (Q1-Q3), n, or mean ± SD.

LAA = left atrial appendage; NA = not applicable.

**Figure 2:**
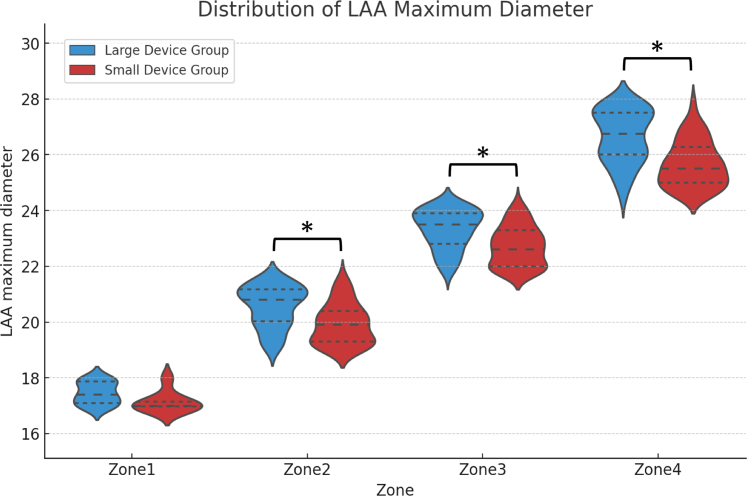
LAA Structural Features Across Zones and Device Groups Violin plots illustrating the scatter distribution of the maximum diameters of the left atrial appendage (LAA) ostium across different zones. ∗*P* < 0.001.

Across all zones, the median number of devices used per patient was consistently low, showing no significant differences between the 2 groups. Both the maximum and minimum LAA diameters were significantly larger in the large device group than in the small device group in zones 2, 3, and 4. LAA depth was not significantly different between the 2 groups across all zones. In addition, the appendage eccentricity index was significantly higher in the small device group than in the large device group (0.18 [Q1-Q3: 0.12-0.25] vs 0.20 [Q1-Q3: 0.14-0.27]; *P* = 0.002). The final device maximum compression rate was significantly higher in the large device group than in the small device group (21.22% ± 4.34% vs 19.63% ± 4.28%; *P* < 0.001). Similarly, the final device minimum compression rate was significantly higher in the large device group than in the small device group (15.86% ± 4.02% vs 14.51% ± 3.50%; *P* < 0.001). Figure 2 presents violin plots depicting the scatter distribution of LAA maximum diameters for each zone.

### Procedural outcomes and periprocedural complications

As shown in Table 3, procedural time (50.14 ± 30.77 vs 52.22 ± 23.90 minutes; *P* = 0.340) and need for redo trans-septal puncture (1.03% [4 of 390] vs 1.63% [6 of 367]; *P* = 0.535) were comparable between the 2 groups. No significant differences were found in the technical success rates (97.44% [380 of 390] vs 99.18% [364 of 367]; *P* = 0.117) or procedural success rates (96.15% [375 of 390] vs 98.37% [361 of 367]; *P* = 0.103) between the large and small device groups.

**Table 3:** Procedural Outcomes and Periprocedural Complications

	Overall (N = 757)	Large Device Group (n = 390)	Small Device Group (n = 367)	*P* Value
General anesthesia	756 (99.87)	390 (100.00)	366 (99.73)	0.485
Procedure time, min	51.15 ± 27.64	50.14 ± 30.77	52.22 ± 23.90	0.340
Technical success	744 (98.28)	380 (97.44)	364 (99.18)	0.117
Procedural success	736 (97.23)	375 (96.15)	361 (98.37)	0.103
Repuncture of the fossa	10 (1.32)	4 (1.03)	6 (1.63)	0.535
Transfusion during the operation	3 (0.40)	0 (0.00)	3 (0.82)	0.114
Residual peridevice leak	24 (3.17)	6 (1.54)	18 (4.90)	0.015
Device embolization	0 (0.00)	0 (0.00)	0 (0.00)	NA
Cardiac tamponade	0 (0.00)	0 (0.00)	0 (0.00)	NA
Pericardial effusion	4 (0.53)	3 (0.77)	1 (0.27)	0.625
Atrial septal injury requiring closure	0 (0.00)	0 (0.00)	0 (0.00)	NA
Conversion to open cardiac surgery	0 (0.00)	0 (0.00)	0 (0.00)	NA
DRT	0 (0.00)	0 (0.00)	0 (0.00)	NA
AKI	0 (0.00)	0 (0.00)	0 (0.00)	NA
TIA	1 (0.13)	0 (0.00)	1 (0.27)	0.485
Major bleeding event	0 (0.00)	0 (0.00)	0 (0.00)	NA
Minor bleeding event	5 (0.66)	4 (1.03)	1 (0.27)	0.375
Vascular complication	0 (0.00)	0 (0.00)	0 (0.00)	NA
In-hospital all-cause mortality	1 (0.13)	0 (0.00)	1 (0.27)	0.485
In-hospital CVA event	0 (0.00)	0 (0.00)	0 (0.00)	NA

Values are n (%) or mean ± SD.

AKI = acute kidney injury; CVA = cerebrovascular accident; DRT = device-related thrombus; TIA = transient ischemic attack.

Periprocedural complications were extremely rare in both groups, and no cases of cardiac tamponade, device embolization, or need for emergency surgery were reported. However, the incidence of residual PDL, evaluated intraoperatively on TEE, was significantly lower in the large device group than in the small device group (1.54% [6 of 390] vs 4.90% [18 of 367]; *P* = 0.015).

### Temporal changes in antithrombotic therapy following LAAC

**Figure 3:**
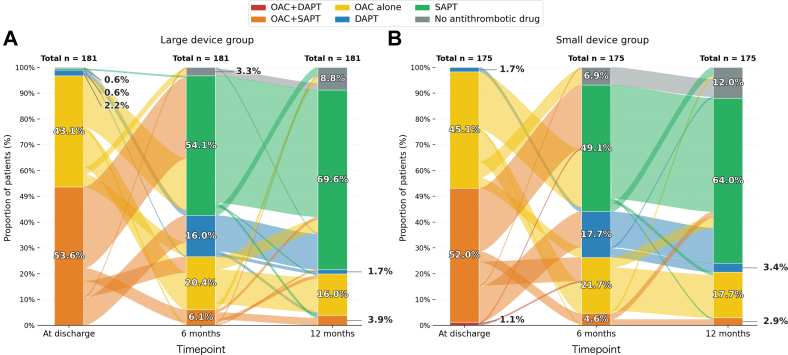
Temporal Changes in Antithrombotic Therapy Changes in antithrombotic therapy over time were analyzed among patients with complete medication records at discharge, 6 months, and 12 months following implantation of the second-generation left atrial appendage closure device. (A) Distribution of antithrombotic regimens in the large device group. (B) Distribution of antithrombotic regimens in the small device group. DAPT = dual antiplatelet therapy; OAC = oral anticoagulation; SAPT = single antiplatelet therapy.

Changes in antithrombotic therapy over time were assessed for paired patient samples with complete medication data at discharge, 6 months, and 12 months after LAAC device implantation (Figure 3). At discharge, oral anticoagulation (OAC) plus single antiplatelet therapy (SAPT) was the most common regimen (large device group, 53.6% [97 of 181]; small device group, 52.0% [91 of 175]), followed by OAC alone (large device group, 43.1% (78 of 181); small device group, 45.1% [79 of 175]). At 6 months, the predominant regimen had shifted to SAPT alone (large device group, 54.1% [98 of 181]; small device group, 49.1% [86 of 175]). OAC-containing regimens (OAC alone and OAC plus SAPT) accounted for 26.5% (48 of 181) and 26.3% (46 of 175) in the large and small device groups, respectively. At 12 months, SAPT alone remained the most frequent regimen (large device group, 69.6% [126 of 181]; small device group, 64.0% [112 of 175]), whereas no antithrombotic therapy was reported in 8.8% [16 of 181] and 12.0% [21 of 175], respectively. OAC-containing regimens further declined to 19.9% (36 of 181) and 20.6% (36 of 175) in the large and small device groups, respectively.

### Postprocedural clinical outcomes

**Figure 4:**
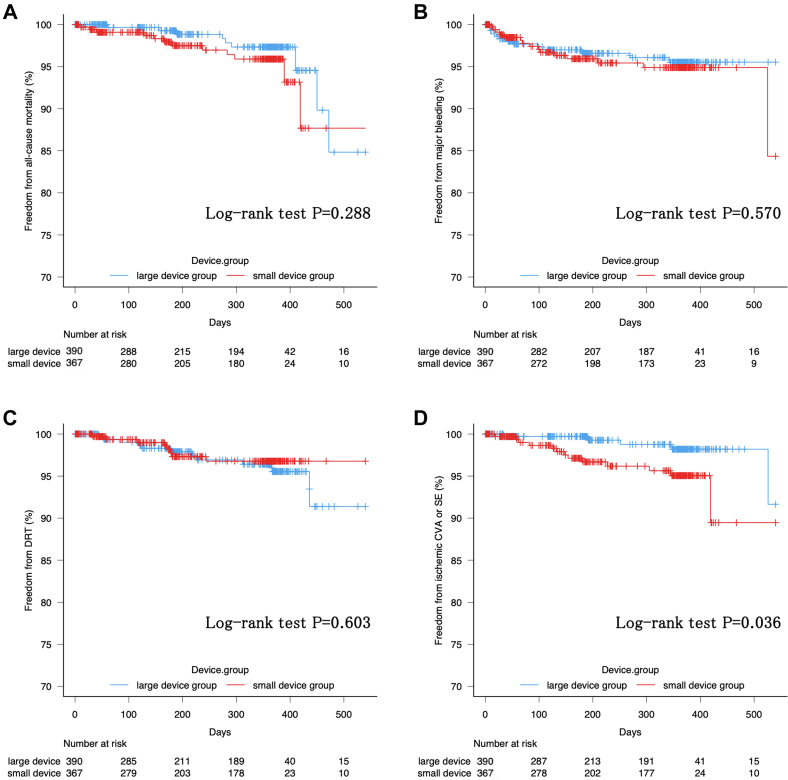
Kaplan-Meier Curves for Clinical Outcomes by Device Size Kaplan-Meier survival curves comparing outcomes between the large and small device groups for freedom from (A) all-cause mortality, (B) major bleeding, (C) device-related thrombus (DRT), and (D) ischemic cerebrovascular accident (CVA) or systemic embolism (SE). The number at risk for each group is displayed below each panel. Log-rank test *P* values are shown for each comparison.

The median follow-up duration of the study cohort was 274 days (Q1-Q3: 100-368 days), with a maximum follow-up duration of 540 days. Kaplan-Meier survival analysis was performed to compare the cumulative event rates for all-cause mortality, ischemic CVA or systemic embolism, major bleeding events, and DRT between the 2 groups (Figure 4). No significant difference was found in all-cause mortality between the large and small device groups (15.2% [95% CI: 6.0%-35.7%] vs 12.3% [95% CI: 4.6%-30.6%]; *P* = 0.288). Similarly, the cumulative incidence rates of major bleeding events (4.5% [95% CI: 2.6%-7.7%] vs 15.7% [95% CI: 4.3%-48.7%]; *P* = 0.570) and DRT (8.6% [95% CI: 3.2%-22.2%] vs 3.2% [95% CI: 1.6%-6.4%]; *P* = 0.603) were comparable between the 2 groups throughout the follow-up period.

However, the cumulative probability of ischemic CVA or systemic embolism was significantly higher in the small device group than in the large device group (10.5% [95% CI: 3.6%-28.4%] vs 8.4% [95% CI: 1.8%-34.1%]; *P* = 0.036).

The Cox univariate regression analysis identified several significant predictors of ischemic CVA or systemic embolism (Table 4). Prior ischemic stroke (HR: 3.84; 95% CI: 1.37-10.79; *P* = 0.011), left ventricular ejection fraction (HR: 0.96; 95% CI: 0.93-1.00; *P* = 0.042), and small device (HR: 2.87; 95% CI: 1.02-8.08; *P* = 0.045) were associated with a higher risk for ischemic CVA or systemic embolism.

**Table 4:** Univariate Cox Regression Analyzing Association Between Cumulative Ischemic CVA or Systemic Embolism and Clinical Findings

	HR	95% CI	*P* Value
Age	0.97	0.92-1.03	0.300
Male	1.92	0.63-5.85	0.251
Permanent or persistent AF	1.47	0.55-3.92	0.443
CHADS_2_ score	1.38	0.97-1.98	0.077
CHA_2_DS_2_-VASc score	1.21	0.89-1.63	0.225
HAS-BLED score	1.29	0.81-2.06	0.284
Hypertension	1.66	0.38-7.21	0.501
Diabetes mellitus	1.47	0.58-3.72	0.418
Prior ischemic stroke	3.84	1.37-10.79	0.011
Prior systemic embolization	2.51	0.72-8.77	0.150
Any cancers	0.47	0.11-2.06	0.318
Steroid use	1.61	0.21-12.13	0.646
LVEF	0.96	0.93-1.00	0.042
eGFR	0.99	0.97-1.01	0.371
LAA maximum diameter	1.04	0.87-1.24	0.665
LAA minimum diameter	0.94	0.80-1.09	0.399
LAA eccentricity index	16.84	0.22-1293.87	0.202
Maximum compression rate	1.10	0.97-1.24	0.145
Minimum compression rate	1.05	0.95-1.16	0.318
Residual peridevice leak	4.07	0.94-17.74	0.061
Small device group	2.87	1.02-8.08	0.045

*P* < 0.05 were considered to indicate statistical significance.

Abbreviations as in Tables 1, 2, and 3.

## Discussion

In this study we evaluated the effect of the second-generation LAAC device sizes on clinical outcomes in patients with borderline-sized LAAs, reflecting real-world clinical practice and providing insights into optimizing device choice for patient care.

First, in real-world clinical practice, a larger ostial diameter was associated with preference for a larger device, whereas a greater LAA eccentricity index was linked to smaller device selection. Second, the large device group demonstrated procedural safety comparable with that of the small device group, with no significant increase in procedural complications. In addition, the risk for DRT did not increase in the large device group, further supporting its safety profile. Third, residual PDLs were significantly less frequent in the large device group than in the small device group. Finally, selecting a larger device was associated with a lower incidence of ischemic CVA or systemic embolism (Central Illustration).

**Central Illustration:**
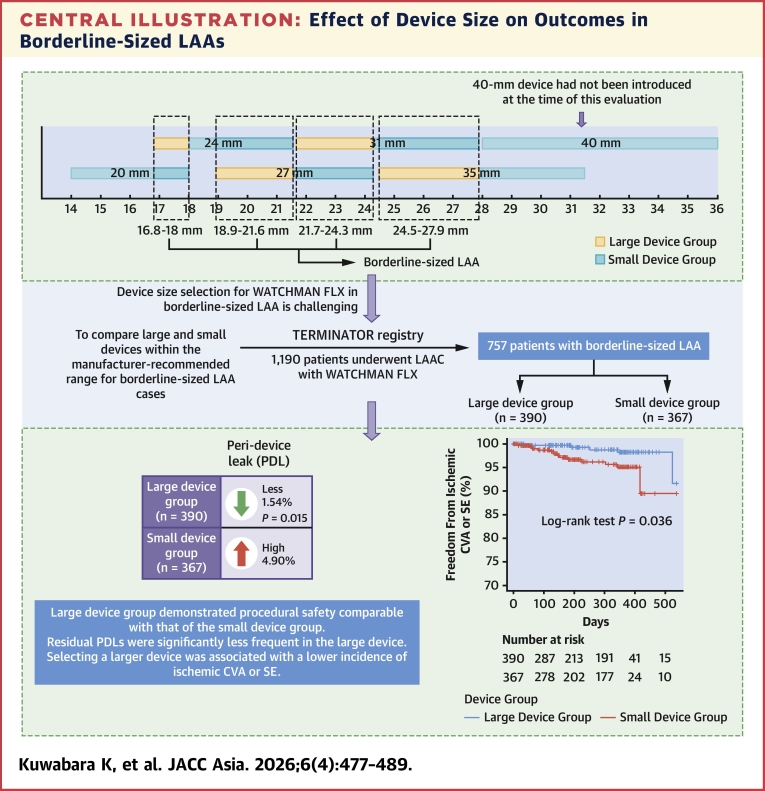
Effect of Device Size on Outcomes in Borderline-Sized LAAs In patients with borderline-sized left atrial appendage (LAA) anatomy, in which 2 manufacturer-recommended device sizes were suitable, the large device group had fewer peridevice leaks (1.54% [6 of 390] vs 4.90% [18 of 367]; *P* = 0.015) and a lower incidence of ischemic cerebrovascular accident or systemic embolism (8.4% [95% CI: 1.8%-34.1%] vs 10.5% [95% CI: 3.6%-28.4%]; *P* = 0.036) than the small device group, without increased procedural complications. CVA = cerebrovascular accident; LAA = left atrial appendage; LAAC = left atrial appendage closure; PDL = peridevice leak; SE = systemic embolism.

### Safety of large devices in borderline-sized LAAs

The potential effect of device sizing on perioperative complications was previously reported using the first-generation LAAC device.^13^ The study showed that selecting a device larger than the manufacturer’s recommendation did not increase the incidence of cardiac tamponade or device embolization. Moreover, current-generation LAAC devices have a significantly improved safety profile, as evidenced by data from the PINNACLE FLX (Protection Against Embolism for Nonvalvular AF Patients: Investigational Device Evaluation of the WATCHMAN FLX LAA Closure Technology) trial^19^ and the National Cardiovascular Data Registry.^20^ Consistent with these findings, the present study, specifically focused on patients treated with the second-generation LAAC device, showed that large devices did not lead to a significant increase in perioperative risks.

Device embolization is a rare but critical complication, with reported rates varying across studies.^19^^,^^21^^,^^22^ A study indicated that smaller devices and minimal differences between the LAA ostial diameter and device size were associated with an increased risk for device embolization.^23^ Although the selection of a larger device has not been explicitly reported as a risk factor for device embolization, cases with inadequate LAA depth may have a higher likelihood of device protrusion when a larger device is selected, potentially leading to device embolization. Notably, the second-generation device is shorter than the first-generation device, providing more opportunities to select a large device without increasing the risk for device embolization. Our findings also indicate that larger devices do not increase the risk for DRT in borderline-sized LAAs. This finding further supports the safety of selecting a larger device when anatomically appropriate.

### PDLs and device selection in borderline-sized LAAs

PDLs are a well-established risk factor for postprocedural thromboembolism.^11^^,^^12^^,^^19^ In the PINNACLE FLX trial, small (0- to 5-mm) leaks following the second-generation LAAC device implantation were reported in 7.4% immediately after procedure and 17.2% at 45 days, whereas large (>5-mm) leaks were not observed.^19^ In this study, PDLs were significantly less frequent in the large device group. Another study showed that devices with sizes beyond the manufacturer’s recommended range significantly reduced PDLs.^13^ Our findings support these observations, demonstrating that even within the manufacturer-recommended size range, the large device group exhibited a lower PDL rate in borderline-sized LAAs.

A recent study also reported positive remodeling of the LAA observed at 6 to 8 weeks after LAAC device implantation, resulting in a decrease in the compression rate during this phase.^24^ The residual compression rate in the subacute phase was significantly lower in cases with persistent LAA patency observed on computed tomography (CT), indicating that achieving adequate compression during implantation may be crucial for ensuring optimal LAA sealing in the subsequent phase.

### Clinical outcomes of large device selection

Selecting the larger device for borderline-sized LAAs significantly reduced the incidence of ischemic CVA or systemic embolism. A study from the National Cardiovascular Data Registry revealed that small (0- to 5-mm) leaks at 45 days were significantly associated with increased risks for stroke, transient ischemic attack, or systemic embolism (HR: 1.152; 95% CI: 1.048-1.160).^11^ A meta-analysis conducted under U.S. Food and Drug Administration oversight, incorporating data from the PROTECT AF (WATCHMAN Left Atrial Appendage System for Embolic Protection in Patients With Atrial Fibrillation), PREVAIL (Evaluation of the WATCHMAN LAA Closure Device in Patients With Atrial Fibrillation Versus Long Term Warfarin Therapy), and CAP2 (Continued Access to PREVAIL) trials, reported that leaks ≤5 mm at 1 year were significantly associated with an increased 5-year risk for ischemic stroke or systemic embolism (HR: 1.94; 95% CI: 1.15-3.29).^12^ In line with the results of previous studies, the findings of the present study suggest that the low incidence of ischemic CVA or systemic embolism observed in the large device group may be attributed to fewer PDLs.

Our results indicate that in cases in which LAA ostial diameters fall within the borderline range for 2 device sizes, the larger device may be preferred because of its safety profile and association with reduced PDLs and lower risk for thromboembolic events. These findings have important implications for clinical practice, as they offer guidance for optimal device selection in patients in whom the best size choice is uncertain.

### Study limitations

First, as a multicenter observational registry study, selection bias is possible, and unmeasured confounders may have influenced the results. Second, long-term follow-up data beyond the study period are needed to confirm the stability of the observed benefits.

Third, the study was conducted exclusively in a Japanese population, so the results may not be fully generalizable to other patient populations with different LAA morphologies. Another limitation is the absence of a formal inter- or intraobserver reproducibility assessment for LAA measurements.

Additionally, no independent core laboratory adjudication was conducted for imaging or outcome events. All measurements and classifications were conducted locally, which may have introduced variability despite the use of standardized registry definitions.

Finally, CT was not mandatory in this registry, and computed tomographic data were insufficient for reliable analysis. Therefore, device selection and residual PDL evaluation were based exclusively on intraprocedural TEE rather than CT-based measurements, which may limit the generalizability of our findings.

## Conclusions

In patients with borderline LAA anatomy undergoing implantation of the second-generation LAAC device, choosing the larger of the 2 manufacturer-recommended device sizes was associated with fewer PDLs, without an increase in procedural complications, ultimately resulting in lower ischemic CVA or systemic embolism rates. These findings provide valuable insights for optimizing device selection strategies. To improve patient outcomes in LAAC procedures, future prospective studies and randomized trials are warranted.

## Funding Support and Author Disclosures

This registry was supported by a grant from Structure Club Japan, a nonprofit organization. Drs Hara, Fukunaga, Kubo, Nakajima, Matsumoto, Kondo, and Kuwabara have received lecture and/or consultancy fees from Boston Scientific outside the submitted work. All other authors have reported that they have no relationships relevant to the contents of this paper to disclose.
